# Imunocompetent Mice Model for Dengue Virus Infection

**DOI:** 10.1100/2012/525947

**Published:** 2012-05-15

**Authors:** Denise Gonçalves, Rafael de Queiroz Prado, Eric Almeida Xavier, Natália Cristina de Oliveira, Paulo Marcos da Matta Guedes, João Santana da Silva, Luiz Tadeu Moraes Figueiredo, Victor Hugo Aquino

**Affiliations:** ^1^Departamento de Análises Clínicas, Toxicológicas e Bromatológicas da Faculdade de Ciências Farmacêuticas de Ribeirão Preto, Universidade de São Paulo, Avenida Bandeirantes 3900, Monte Alegre, 14049-900 Ribeirao Preto, SP, Brazil; ^2^Centro de Pesquisa em Virologia da Faculdade de Medicina de Ribeirão Preto, Universidade de São Paulo, Avenida Bandeirantes 3900, Monte Alegre, 14049-900 Ribeirao Preto, SP, Brazil; ^3^Departamento de Microbiologia e Parasitologia, Universidade Federal do Rio Grande do Norte, Avenida Salgado Filho SN, Campus Universitário, 59078-900 Natal, RN, Brazil; ^4^Departamento de Parasitologia, Microbiologia e Imunologia da Faculdade de Medicina de Ribeirão Preto, Universidade de São Paulo, Avenida Bandeirantes 3900, Monte Alegre, 14049-900 Ribeirao Preto, SP, Brazil

## Abstract

Dengue fever is a noncontagious infectious disease caused by dengue virus (DENV). DENV belongs to the family *Flaviviridae*, genus *Flavivirus*, and is classified into four antigenically distinct serotypes: DENV-1, DENV-2, DENV-3, and DENV-4. The number of nations and people affected has increased steadily and today is considered the most widely spread arbovirus (arthropod-borne viral disease) in the world. The absence of an appropriate animal model for studying the disease has hindered the understanding of dengue pathogenesis. In our study, we have found that immunocompetent C57BL/6 mice infected intraperitoneally with DENV-1 presented some signs of dengue disease such as thrombocytopenia, spleen hemorrhage, liver damage, and increase in production of IFN**γ** and TNF**α** cytokines. Moreover, the animals became viremic and the virus was detected in several organs by real-time RT-PCR. Thus, this animal model could be used to study mechanism of dengue virus infection, to test antiviral drugs, as well as to evaluate candidate vaccines.

## 1. Introduction

 Dengue is a noncontagious infectious disease caused by dengue virus (DENV), which is a positive-sense, single-stranded RNA virus that belongs to the family *Flaviviridae*, genus *Flavivirus*. DENV is classified into four antigenically distinct serotypes: DENV-1, DENV-2, DENV-3, and DENV-4 [1–3]. The number of nations and people affected has increased steadily and today is considered the most widely spread arbovirus (arthropod-borne viral disease) in the world. An estimated 50 million dengue infections occur annually and approximately 2.5 billion people live in dengue endemic countries [4]. Dengue can be presented in three main clinical forms: undifferentiated febrile illness, dengue fever (DF), and dengue hemorrhagic fever (DHF) with or without shock syndrome known as dengue shock syndrome (DSS). The DF is presented as a self-limiting acute febrile illness that lasts for about 4 to 5 days. The disease begins abruptly with high fever, retroorbital pain, headaches of varying degrees, maculopapular rash, muscle and joint aches, and can also be observed mild hemorrhagic phenomena. In DHF/DSS, in addition to the initial symptoms corresponding to DF, between the second and fourth days of infection, there is an increased thrombocytopenia and bleeding phenomena, abdominal pain, and leakage of fluid into the interstitium, which can lead to decrease intravascular plasma volume and consequent hypovolemic shock that in some cases could be fatal [5].

 The absence of an appropriate animal model for studying the disease has hindered the understanding of dengue pathogenesis. Several studies using immunocompetent, immunocompromised and humanized mice have been developed to analyze various aspects of the disease [6–12]. These experimental models are useful according to the variable of the disease to be evaluated.

 The main objective of our study was to evaluate the C57BL/6 mice as a model of dengue virus infection.

## 2. Material and Method

### 2.1. Mice

 Three-to-four-week-old C57BL/6 mice were obtained from the Central Animal Facility at the University of Sao Paulo/Ribeirao Preto and housed in specific pathogen free (SPF) conditions at the Research Center of Virology, Medical School of Ribeirao Preto (FMRP). All the procedures were approved by the Animal Ethics Committee of the FMRP (Protocol: 103/2005).

### 2.2. Virus

 DENV-1, Mochizuki strain, was used in this study. The virus was maintained in C6/36 cells culture at 28°C for 7 days. Then, the virus was propagated in newborn (1 to 2 days old) Swiss mice, by intracerebral inoculation of infected cell culture supernatant. After the first appearance of signs of paralysis, mice were sacrificed and stored at −70°C until use. The brains of infected and uninfected animals were removed with sterile syringes and prepared as a suspension of 20%, using PBS containing 4% bovine fetal serum. The brain was then macerated and the suspension was centrifuged at 10,000 ×g for five minutes. The supernatant was aliquoted and stored at −70°C. The viral title was determined by the plaque assay [6].

### 2.3. Animal Infection and Sample Processing

 The animals were infected intraperitoneally with 150 *μ*L viral suspension containing 7.2 × 10^7^ PFU or 150 *μ*L of uninfected brains suspension. At different times postinfection, the blood was obtained from the retroorbital region and collected in a tube containing sodium citrate (3.8%) as an anticoagulant in an amount corresponding to 10% of the total volume; then, the animals were sacrificed to remove liver, brain, spleen and kidney. The blood was centrifuged at 1000 ×g for five minutes to obtain the plasma. The whole organs were suspended in 500 mL of PBS and ground in a tissue homogenizer, except liver that only the right lobe was used. The suspension was centrifuged at 8000 ×g for five minutes and the supernatant was stored at −70°C until use for viral load quantification. Liver and brain of four mice were fixed in 4% neutral formalin solution, embedded in paraffin, and sectioned at a thickness of 3 *μ*m. After deparaffinization and rehydration, the sections were stained with hematoxylin and eosin (H&E). The sections were then dehydrated before mounting. The total number of nucleated cells was counted in fifty microscopic fields in at least four representative, nonconsecutive, HE-stained sections from each mouse. Sections were examined using a Zeiss Integrationsplatte II eyepiece (Zeiss Co, Oberkochen, Germany) reticule, using a microscope at a final magnification of 400x. For hepatic steatosis observation; liver of four mice was embedded in Tissue-Teck OCT (Sakura Finetek, USA) and snap frozen in isopentane cooled in liquid nitrogen and stored at −80°C. Cryosections of 5 *μ*m thickness were mounted on a glass slide, fixed in acetone for 5 minutes and stained with hematoxylin and Sudan III.

### 2.4. Viral Quantification

 The RNA was purified from 140 *μ*L of plasma or supernatant of the organs suspensions using the QIAamp Viral RNA Mini Kit (QIAGEN, Germany) according to the protocol recommended by the manufacturer. The viral RNA was eluted with 80 *μ*L of distilled water free of DNase/RNase and stored at −70°C. The real-time RT-PCR was carried out as described previously [7]. A standard curve was constructed using a decimal serial dilution of RNA obtained form the viral seed (4.8 × 10^8^ PFU/mL). The viral title in plasma/organs was expressed as PFU/mL.

### 2.5. Hematocrit

 The blood was collected into tubes containing anticoagulant and transferred to a capillary microhematocrit (Perfecta, Brazil). The capillaries were centrifuged for 5 minutes at 10,000 ×g in a microcentrifuge (Quimis, Brazil). The analysis was performed according to manufacturer's recommendation.

### 2.6. Platelets

 Platelet count was determined by the method of Brecher and Cronkite [13]. 

### 2.7. Liver Enzymes

 Oxaloacetic transaminase (AST) and pyruvic transaminase (ALT) levels were measured in the plasma of infected and not infected mice by specific biochemical test (Labtest, Brazil) using Kinetic UV-IFCC methodology, according to the manufacturer's recommendations.

### 2.8. Spleen Cells Identification

 The spleen of each mouse was removed and placed in a small petri dish containing 3 mL of sterile incomplete RPMI culture medium, on ice. Then, the organ was macerated to obtain a cell suspension, which was centrifuged at 1000 ×g for 10 min at 4°C. The supernatant was discarded and the pellet was suspended in 4 mL of erythrocyte lysis buffer ACK (0,15 M of NH_4_Cl, KHCO_3_, and 0,1 mM of EDTA) and incubated at room temperature for 4 minutes. Then, 6 mL of complete RPMI (with 10% of fetal bovine serum) was added to the cells suspension and centrifuged at 1000 ×g for 10 min at 4°C. The supernatant was discarded and cells were suspended in 1 mL of complete RPMI medium for subsequent counting in a Neubauer chamber. Aliquots of 100 *μ*L of suspension containing 1 × 10^6^ spleen cells were incubated with 100 *μ*L of 5% rabbit serum for 40 minutes to minimize the possibility of unspecific binding of monoclonal antibodies (to block Fc and non-specific Ig binding sites) to cell surface molecules. Then, samples were incubated with 1 *μ*L of surface markers monoclonal antibodies (anti-CD3, anti-CD4, anti-CD8, anti-CD11b, anti-CD11c, anti-CD25, and anti-CTLA-4) or isotype controls (IgG_1_-FITC, IgG_2a_-PE, IgG1-IgG_1_-PerCP, and IgG_2a_-APC) directly conjugated to FITC, PE, PerCP, and/or APC fluorochromes (Becton-Dickinson, USA, or PharMingen, USA) for 30 minutes at room temperature, in the dark. The cells were centrifuged for 5 minutes at 1000 ×g, washed twice with PBS, suspended in 200 *μ*L of PBS, and analyzed immediately on the FACSort flow cytometer (Becton-Dickinson, USA), acquiring a minimum of 100,000 events. Two or three antibodies were used at the same time to mark the cellular subpopulations of the spleen. The following subcell populations present in the spleen were analyzed: CD3^+^ (T lymphocytes), CD3^+^ CD4^+^ (T helper cells), CD3^+^ CD8^+^ (cytotoxic T cells), CD11c^−^ CD11b^+^ (macrophages), CD11c^+^ CD11b^−^ (dendritic cells), and CD4^+^ CD25^+^ CTLA-4^+^ (regulatory T cells).

### 2.9. Spleen Cell Culture for Detection of Intracellular Cytokines

 The spleen of each animal was macerated and the cells suspension was adjusted to 5 × 10^6^ spleen cells/mL with RPMI 1640 containing 10% FBS. This suspension was incubated at 37°C for 48 hours. Then, PMA (10–50 ng/mL) and ionomicyn (100–500 ng/mL) were added and the suspension was vortexed. The cells were centrifuged at 1000 ×g for five minutes and then washed with cold PBS. The cells were incubated with 0.5 *μ*g of anti-CD16/CD32 mAb (FC receptor blocking) at 4°C for 30 minutes and then 0.5 *μ*g of FITC-or PerCP labeled anti-CD3, CD4, and CD8 (BD PharMingen, San Diego, CA, USA) were added and incubated for 30 min at 4°C in the dark. For the detection of intracellular IL-17, IL-10, TNF-*α*, and IFN-*γ* cytokines, the subpopulation of CD4 cells was fixed and permeabilize using Cytofix/Cytoperm (BD Biosciences, USA), following manufacturer's recommendations. Then, the cells were washed and incubated with anti-IL-17, anti-IL-10, anti-TNF-*α*, and anti-IFN-*γ* conjugated with FITC-, APC-, or PE-fluorochromes, overnight at 4°C in the dark. Then, the cells were washed twice in 200 mL of PBS with 1% formaldehyde and resuspended in the same solution. The cells were immediately analyzed on the FACSort flow cytometer (Becton-Dickinson, USA), acquiring a minimum of 50,000 events, and then the data were analyzed using FlowJo software.

### 2.10. Quantification of Serum Cytokines

 Serum samples were added to tubes containing 50 mg/mL a protease inhibitor cocktail in PBS (Complete, Roche, USA). The samples were centrifuged and the supernatant collected for cytokine quantification. The level of cytokines (IL-10, IFN-*γ*, and TNF-*α*) in serum was measured using an ELISA kit (R&D, Minneapolis, MN, USA) and procedures were in accordance with manufacturers' specifications. The optical density was measured at 450 nm. The results were expressed in picograms per milliliter (pg/mL).

## 3. Results

### 3.1. Viral Detection

**Figure 1:**
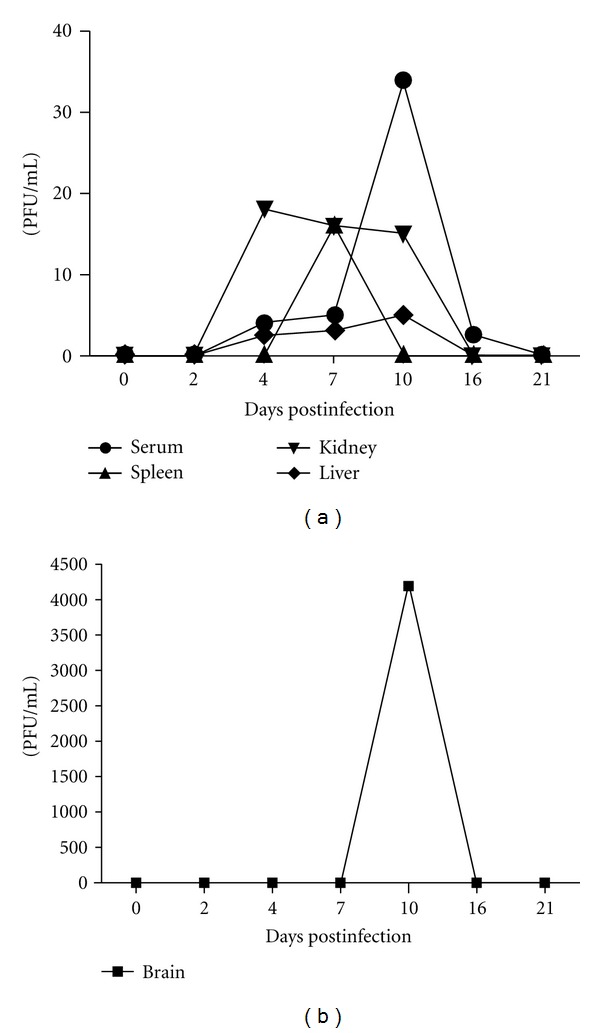
Viral load determined by real time RT-PCR in liver, spleen, kidney, serum (a), and brain (b) of infected animals.

 To analyze whether the mice were susceptible to infection, 24 animals were inoculated intraperitoneally with DENV-1. A group of 4 animals were sacrificed 2, 4, 7, 10, 16, and 21 days after infection for viral load determination in serum, brain, liver, kidney, and spleen. Viral RNA was detected by real-time RT-PCR from the second to the sixteenth day after infection in blood and all analyzed organs (Figure 1). A higher viral load was observed in blood and brain, with a peak on the tenth day after viral infection. These results showed that DENV-1 (Mochizuki strain) inoculated intraperitoneally, established viremia, and disseminated to various organs.

### 3.2. Platelets and Hematocrit

**Figure 2:**
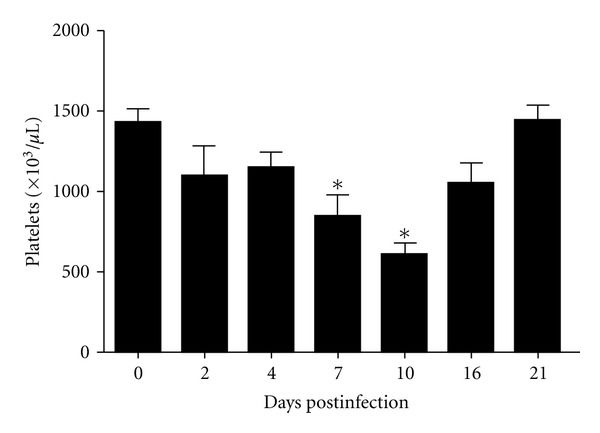
Number of platelets in C57BL/6 along the infection DENV1. The data were analyzed using the Student's *t*-test and differences were considered significant when *P* < 0.05 (*).

 To analyze the platelets and hematocrit after infection, blood sample from the retroorbital region of five mice was obtained 2, 4, 7, 10, 16, and 21 days after infection. The infected mice showed significant reduction of platelet count between seven and ten days after infection, and subsequently the platelet count return to normal levels (Figure 2).

**Figure 3:**
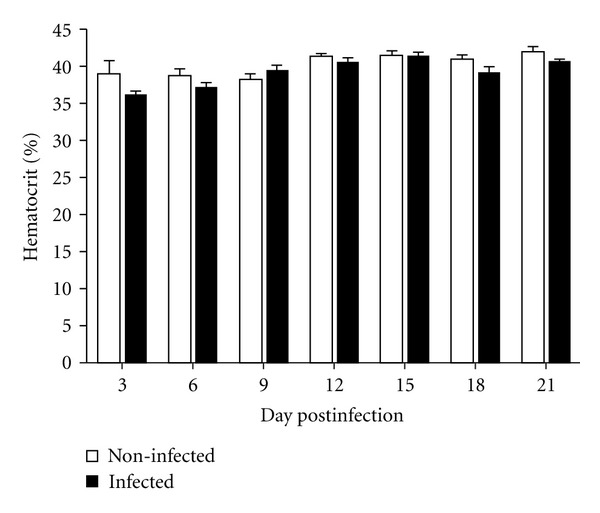
Hematocrit levels of C57BL/6 infected with DENV-1 and uninfected control animals.

 The hematocrit was determined to evaluate the plasma leakage into the interstitium; however, no difference in the hematocrit between control and infected mice was observed (Figure 3).

### 3.3. Analysis of Liver Damage and Cellular Infiltration

**Figure 4:**
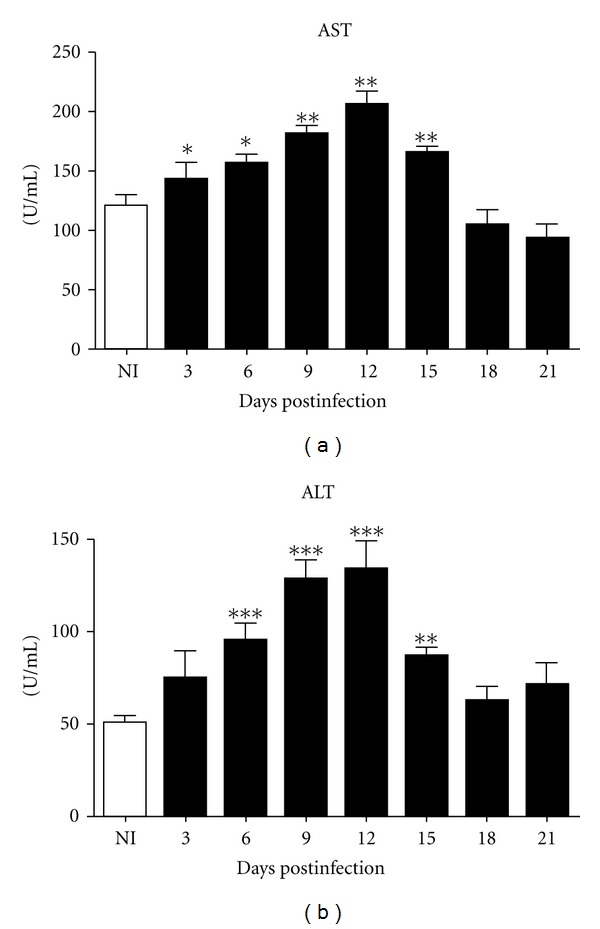
AST (a) and ALT (b) levels in infected and uninfected C57BL/6. Data were analyzed using the Student's *t*-test and differences were considered significant when *P* < 0.05 (*).

To investigate any liver damage induced by infection, oxaloacetic (AST), and pyruvic transaminase (ALT) enzymes were measured in plasma. An increase level of both enzymes was observed after infection, reaching a maximum concentration on day 12 after infection, with a recovered of normal levels on day 18 after infection (Figure 4).

**Figure 5:**
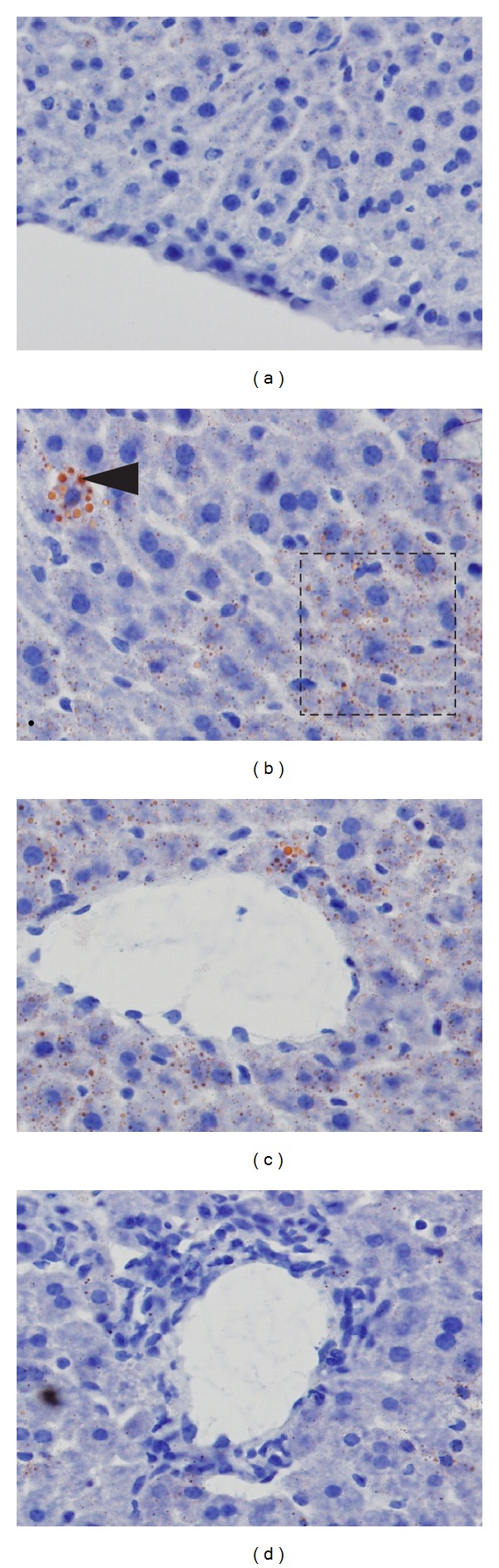
Histology of liver of C57BL/6 on the ninth day of infection with DENV-1 (a). Liver of noninfected mice, showing normal aspect; (b) and (c). Infected mouse liver, showing fat accumulation; (d). Infected mouse liver, showing inflammatory infiltrate. Hematoxylin and Sudan III. The magnification was 400x.

To further analyze the presence of liver damage, five animals were sacrificed nine days after infection and the presence of steatosis in the liver was qualitatively evaluated.  Figure 5 shows the presence of microesteatose in the liver of infected mice, suggesting a metabolic liver disorder. In addition, the presence of inflammatory infiltrate of mononuclear lymphocytes was also observed.

**Figure 6:**
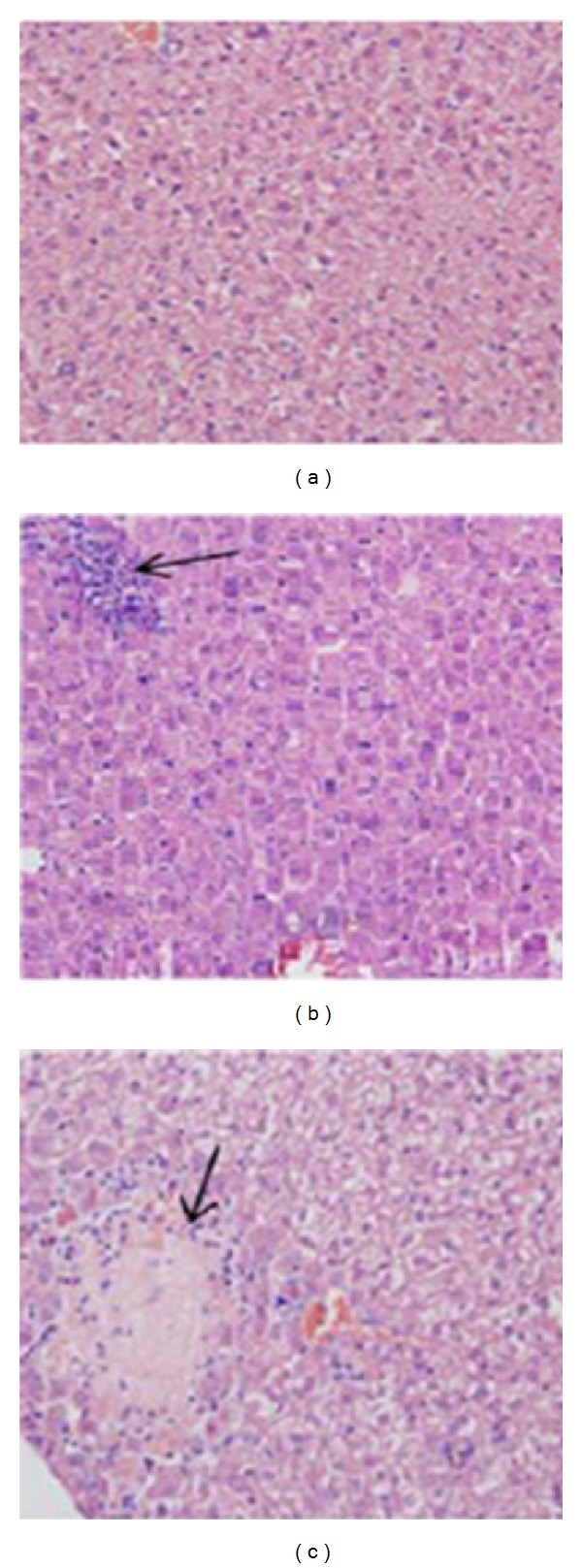
Histology of liver of uninfected mice (a); seven days after infection (b) and 10 days after infection (c). The arrows indicate the presence of cellular infiltrate. Eosin and Hematoxylin staining. The magnification was 200x.

**Figure 7:**
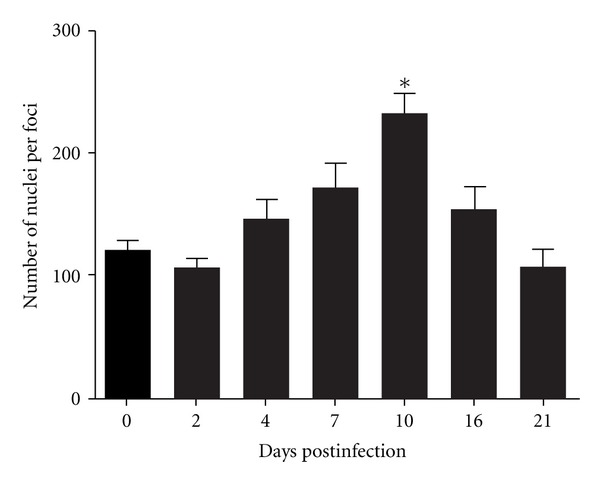
Count of liver nuclei of mice infected with DENV-1. The data were analyzed using the Student's *t*-test and differences were considered significant when *P* < 0.05 (*).

To further investigate the presence of cellular infiltrate in the liver, the animals were infected and 0, 2, 4, 7, 10, 16, and 21 after infection a histological analysis of the liver was carried out. The analysis of the slides showed the presence of cellular infiltrate in the liver, with a significant increase ten days after infection (Figures 6 and 7).

### 3.4. Analysis of Cellular Infiltration in the Brain

**Figure 8:**
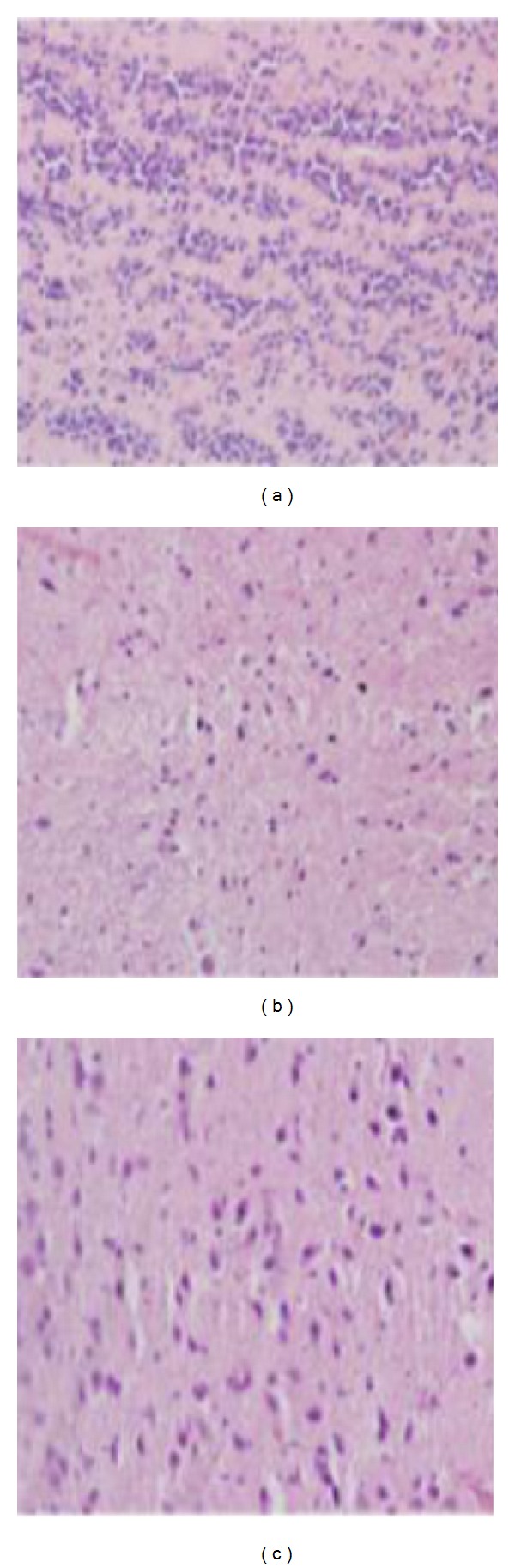
Histology of the animal brains. Uninfected mice (a); mice after seven days of infection (b), and mice after ten days after infection (c). Eosin and hematoxylin staining. The magnification was 200x.

**Figure 9:**
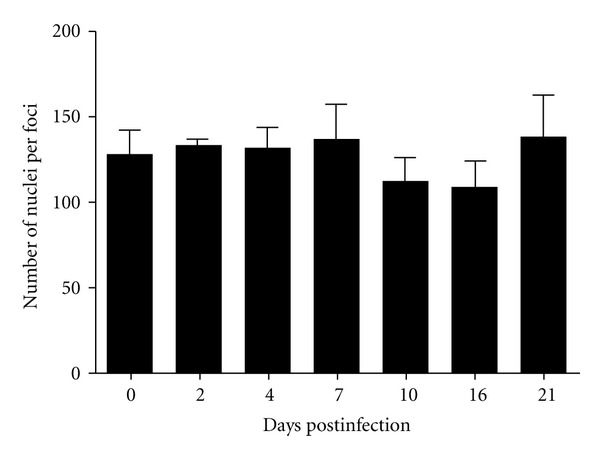
Count of brain nuclei of mice infected with DENV-1. The data not demonstrated a statistically significant difference.

To investigate the presence of cellular infiltrate in the of brain of infected mice, the animals were sacrificed 2, 4, 7, 10, 16, and 21 days after infection to carry out histological analysis. The analysis of the slides showed no cellular infiltration in the brain (Figures 8 and 9).

### 3.5. Morphological Analysis of Spleen

**Figure 10:**
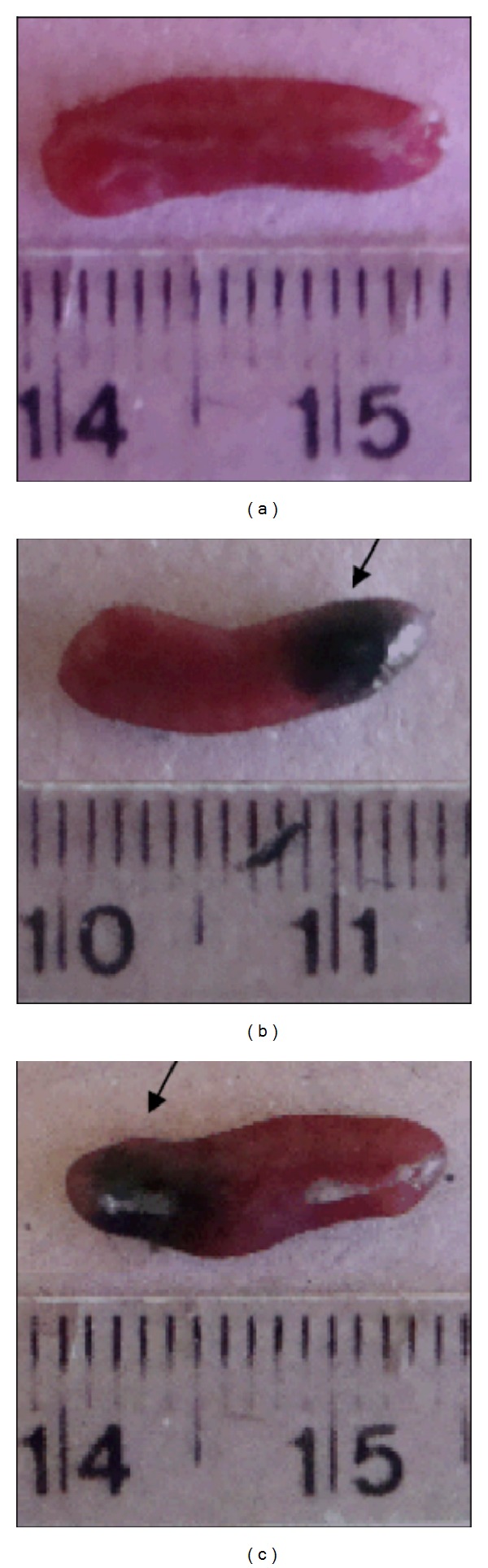
Macroscopic observation of the spleen from uninfected C57BL/6 mice (a), infected and collected 2 (b) and 7 days after infection (c). The arrows indicate points of hemorrhage.

 For analysis of splenic injury, we conducted a morphological analysis and found hemorrhagic spleens in the infected mice (Figure 10).

### 3.6. Spleen Cellular Profile

**Figure 11:**
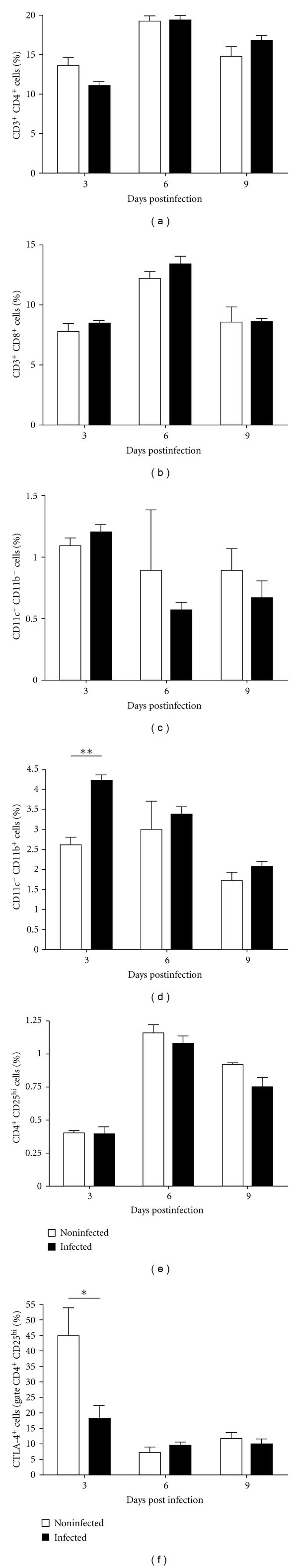
Percentage of CD4^+^ (a), CD8^+^ (b), dendritic cells (c), macrophages (d), and regulatory T cells ((e) and (f)) from the spleen in the third, sixth, and ninth days of infection with DENV1. The data were analyzed using the Student's *t*-test and differences were considered significant when *P* < 0.05 (*).

The phenotype of immune cells in the spleen was investigated by flow cytometry analysis on days 3, 6, and 9 after infection (Figure 11). No significant increase of the number of CD4^+^ and CD8^+^ T lymphocytes and dendritic cells was observed in the animals in any day after infection when compared to the uninfected animal. However, an increase in the population of macrophage (CD11c^−^ CD11b^+^ phenotype) cells was observed in the infected animals three days after infection. Were also determined the percentage of regulatory T cells CD4^+^ CD25hi which showed similar levels between infected and uninfected mice, but this population of cells was an apparent decrease in the expression of CTLA4 on the third day of infection.

### 3.7. Spleen Intralymphocyte CD4^+^ Cytokines Profile

**Figure 12:**
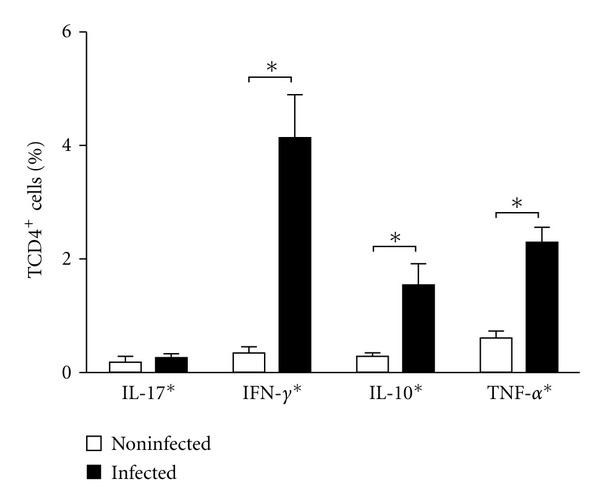
Quantification of intracellular IL17, IFN*γ*, IL-10, and TNF-*α* cytokines in the spleen CD4^+^ cells. The data were analyzed using the Student's *t*-test and differences were considered significant when *P* < 0.05 (*).

Lymphocyte T CD4^+^ cells of infected animals showed a significant production of IL-10, TNF-*α*, and IFN-*γ* cytokines when compared to uninfected animals (Figure 12). On the other hand, no difference was observed between infected and uninfected animals in the production of IL-17.

### 3.8. Cytokine Profile by ELISA in Serum

**Figure 13:**
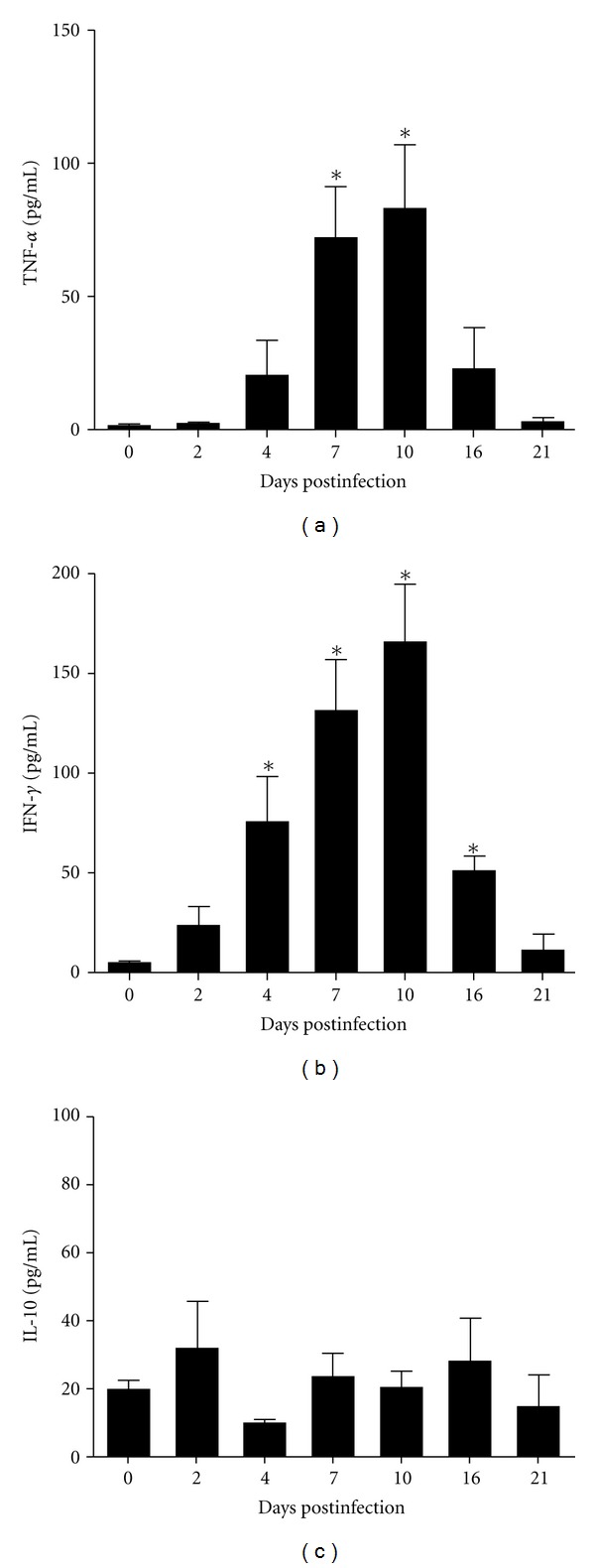
Levels of (a) TNF*α* showed a statistically significant difference at days 7 and 10, (b) IFN*γ* showed a statistically significant difference at days 4, 7, 10, and 16, and (c) IL-10 not demonstrated a statistically significant difference. The data were analyzed using the Student's *t*-test and differences were considered significant when *P* < 0.05 (*).

 Four animals were sacrificed 2, 4, 7, 10, 16, and 21 days after infection to obtain the serum for cytokines quantification (TNF, IFN*γ*, and IL-10). The infected animals showed a significant increase in production of TNF*α* and IFN*γ* compared to the uninfected animals, but no difference was observed between them in the production of IL-10 (Figure 13).

## 4. Discussion

 Symptoms and signs of dengue fever include sudden onset of high fever, retroorbital headache, rash, joint and muscle pain, some degree of hemorrhage, low platelet counts and liver damage. Several immunodeficient animal models have been described to study different aspects of the disease [7, 9, 10, 14]. Several animal models have been used to study the disease, including immunocompetent [6, 8] immunocompromised [7, 9] and humanized mice [15].

 However, it is well known that the immune system combats an invading pathogen by a very complex interplay between the many different components of this system; thus, important interplay between those components can be masked by the immunodeficiency induced in those animals, leading, in some cases, to misleading conclusions. In this study, we have established an experimental model of DENV infection using immunocompetent C57BL/6 mice, which showed some signs of DF similar to those observed in human. Moreover, the animals became viremic and the virus spread to several organs.

Approximately, one-third of DF patients may have mild hemorrhage manifestations, which may be related to the low platelet count [5]. Then, platelet count is a useful marker to be evaluated in an animal model during DENV infection. In that sense, our C57BL/6 mice model showed a significant thrombocytopenia between seven and ten days after DENV-1 infection. In other studies, H. C. Chen, (2007), and Y. T. Yen, (2008), have also shown that C57BL/6 mice infected with DENV-2 showed a significant thrombocytopenia and severe systemic hemorrhage when a very high viral dose (2 × 10^9^ PFU) was used for the infection, but no hemorrhage was observed at lower viral dose (4 × 10^7^ PFU). In our study, we did not analyze in more details the presence of hemorrhage, but macroscopic hemorrhage was observed in the spleen of the infected animals.

After the mosquito biting, human skin dendritic cells (Langerhans cells) are one of the first targets for DENV infection [15–18]. Then, the virus spread systemically infecting several organs. In that sense, DENV has been found in different organs such as skin [19], liver [20–22], spleen [21–23], lymph nodes [21, 22], kidney [22, 24], bone marrow [22, 24], lung [22, 24], thymus [25], and brain [26]. The animal model analyzed in our study showed the presence of viral genome in kidney, liver, spleen and brain after infection. Although liver is not a major target organ, several studies have demonstrated elevated liver enzyme levels in human serum indicating the damage of this organ after DENV infection [27–30]. Hepatitis associated with dengue fever is also characterized by moderate hepatocytes necrosis, microvesicular steatosis and cellular infiltration [31–33]. The C57BL/6 mice analyzed in this study presented high concentration of serum transaminases, liver inflammatory infiltration, and steatosis showing the injury induced by viral infection. H. C. Chen and colleagues have also observed liver damage using the same animal model; however, they infected the animals with a viral dose of 1 × 10^8^ PFU, while in our study we have used a lower viral dose (7.2 × 10^7^ PFU), which is easier to manage in the laboratory [6]. In addition, they used an intravenous route of infection instead of the intraperitoneal route used in our study. DENV infection of animals by peripheral inoculation, such as the intraperitoneal route, has been shown to reproduce some aspect of human disease [8, 12, 34]. The intraperitoneal route can also facilitate the infection of macrophage, which is one of the initial targets of DENV infection [35]. Other studies using BALB/c as animal model have found also liver damage; however, the virus was not detected in serum or detected only after serum inoculation in cell culture [36, 37].

 One of the main components of the immune system against DENV infection includes interferons (IFNs) from the Th1 cytokine profile [38–40]. Shresta and colleagues have shown that AG129 mice lacking both IFN-*α*/*β* and IFN-*γ* receptors are completely susceptible to DEN2-induced disease, showing that these cytokines have critical function resolving DENV infection [41]. H. C. Chen and colleagues have found that C57BL/6 infected with 1 × 10^8^ PFU of DENV-2 strain 16681 showed a significant increase of CD4^+^ and CD8^+^ T cells count and production of IFN*γ* [6]. In our study, although no increase of CD4^+^ and CD8^+^ T cells count was observed, a significant increase of IFN*γ*, with peak on day 10 after infection, was observed infecting the animals with DENV-1 strain Mochizuki with a lower viral dose (7.2 × 10^7^ PFU).

 TNF-*α* has been recognized as an important factor for the development of the severe dengue disease [42–44]. C57BL/6 mice have been established as a dengue hemorrhage model and found that TNF-*α* is a very important cytokine that induces endothelial damage and hemorrhage [7, 45]. These authors used an extremely high viral dose (2 × 10^9^ PFU) to induce systemic hemorrhage and only local subcutaneous hemorrhage when they used a lower viral dose (8 × 10^7^ PFU). Although we did not analyze the hemorrhage in more details, mice infected with a lower viral dose (7.2 × 10^7^ PFU) showed spleen hemorrhage, suggesting a systemic injury.

 IL-10 is an important component of the Th2 cytokine profile that contains and suppresses inflammatory responses [46, 47]. In our study, we have found an increase of spleen CD4^+^ T lymphocytes producing IL-10 seven days after infection, in addition to the IFN*γ* and TNF-*α* producing cells. However, no difference in the serum concentration of IL-10 was found when compared to the uninfected animals, in contrast to the increase concentration of IFN*γ* and TNF-*α* (Figure 13). This discrepancy can be related to the low number of IL-10 producing cells observed in the spleen when compared to the IFN*γ* and TNF-*α* producing cells. In addition, the identification of IL-10 producing cells was observed by *in vitro* polyclonal stimulation and not necessarily reflects the serum concentration.

 Several studies have been demonstrated that spleen cells are target of infection with DENV in humans and mice [21, 22]. Basilio de Oliveira and colleagues showed in a case report that spleen was grossly congested with multiple hemorrhagic foci [21]. We found difference in the size between spleens of infected and uninfected mice and hemorrhagic foci at days 2 and 7 after infection.

 In summary, we have found that C57BL/6 mice infected intraperitoneally with DENV-1 presented some signs of dengue disease such as thrombocytopenia, hemorrhage, liver damage, and increase production of IFN*γ* and TNF*α* cytokines. Thus, this animal model could be used to study dengue virus infection, to test antiviral drugs as well as to candidate vaccines.

##  Authors' Contribution

D. Gonçalves, R. Prado and E. Xavier participated equally to this paper.
