# Corrigendum to “Immunocompetent Mice Model for Dengue Virus Infection”

**DOI:** 10.1155/2018/5268929

**Published:** 2018-09-06

**Authors:** Denise Gonçalves, Rafael de Queiroz Prado, Eric Almeida Xavier, Natália Cristina de Oliveira, Paulo Marcos Da Matta Guedes, João Santana da Silva, Luiz Tadeu Moraes Figueiredo, Victor Hugo Aquino

**Affiliations:** ^1^Departamento de Análises Clínicas, Toxicológicas e Bromatológicas da Faculdade de Ciências Farmacêuticas de Ribeirão Preto, Universidade de São Paulo, Avenida Bandeirantes 3900, Monte Alegre, 14049-900 Ribeirao Preto, SP, Brazil; ^2^Centro de Pesquisa em Virologia da Faculdade de Medicina de Ribeirão Preto, Universidade de São Paulo, Avenida Bandeirantes 3900, Monte Alegre, 14049-900 Ribeirao Preto, SP, Brazil; ^3^Departamento de Microbiologia e Parasitologia, Universidade Federal do Rio Grande do Norte, Avenida Salgado Filho SN, Campus Universitário, 59078-900 Natal, RN, Brazil; ^4^Departamento de Parasitologia, Microbiologia e Imunologia da Faculdade de Medicina de Ribeirão Preto, Universidade de São Paulo, Avenida Bandeirantes 3900, Monte Alegre, 14049-900 Ribeirao Preto, SP, Brazil

In the article titled “Imunocompetent Mice Model for Dengue Virus Infection” [1], there was a minor spelling mistake in the first word in the article's title where “Immunocompetent” was written wrongly as “Imunocompetent.” The correct title is shown above.
